# Supplementary material for: Equation of State of Charged Rod Dispersions
Source: J Phys Chem B. 2023 Oct 13;127(42):9058–65. doi: 10.1021/acs.jpcb.3c04590 (PMC10614191; doi:10.1021/acs.jpcb.3c04590)
Supplement: Supplementary file 1 — jp3c04590_si_001.pdf [file jp3c04590_si_001.pdf]

Supporting Information for Publication Equation of State of Charged Rod Dispersions

Remco Tuinier*,† and Anja Kuhnhold*,‡

†*Laboratory of Physical Chemistry, Department of Chemical Engineering and Chemistry, & Institute for Complex Molecular Systems (ICMS), Eindhoven University of Technology, P.O. Box 513, 5600 MB, Eindhoven, the Netherlands*

‡*Institute of Physics, University of Freiburg, Hermann-Herder-Str. 3, 79104 Freiburg, Germany*

E-mail: r.tuinier@tue.nl; anja.kuhnhold@physik.uni-freiburg.de

Here we show the relative difference between the approximation and the exact expression for A', and some computer simulation results for other rod aspect ratios as those presented in the main text.

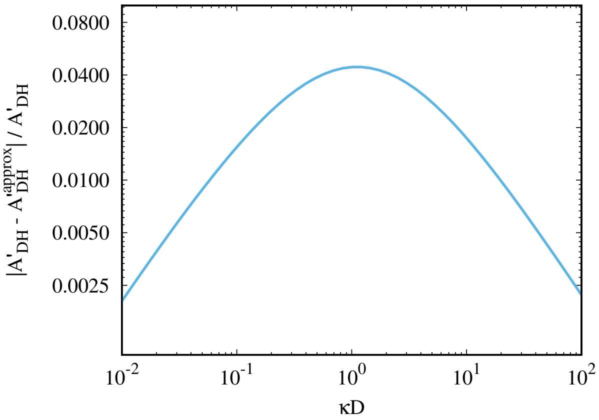

Figure S1: Relative difference between Eqs. (11) and (13) .

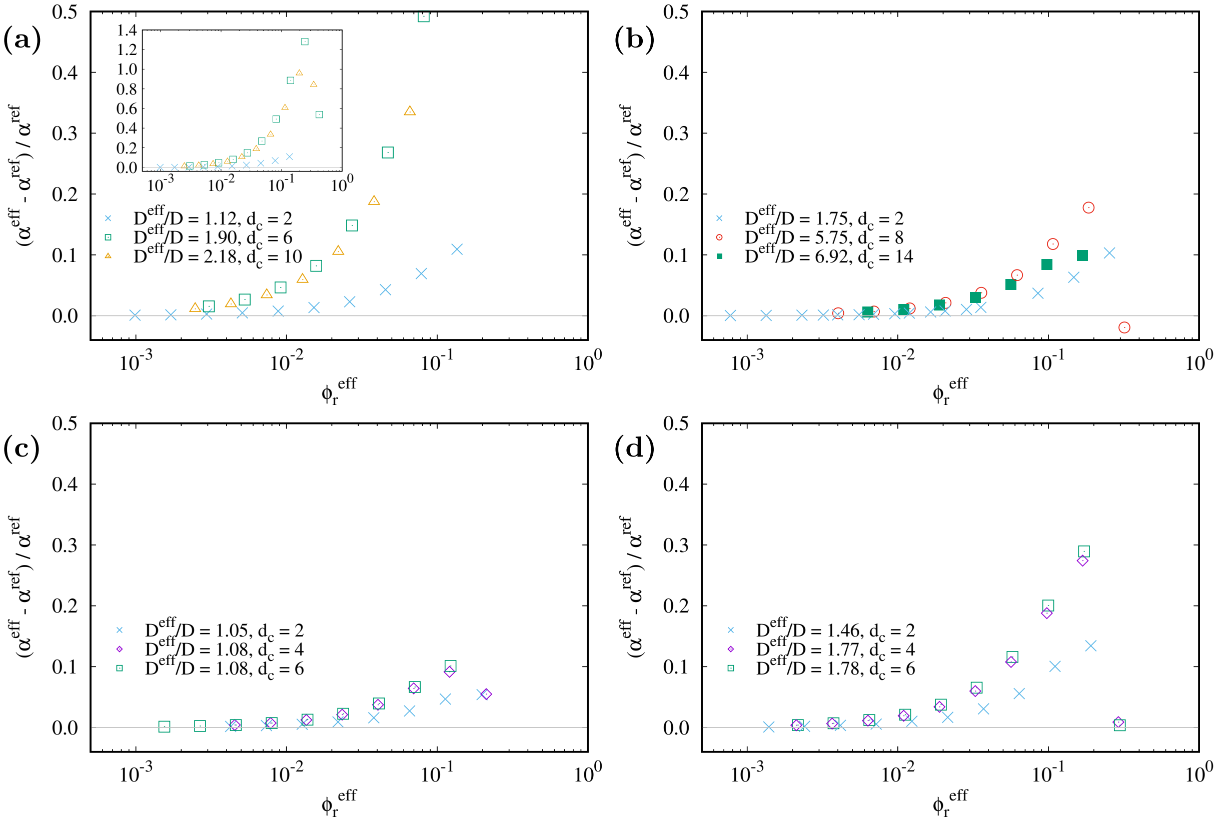

Figure S2: Relative difference between the effective free volume fraction and the reference from the immersion free energy vs. effective volume fraction for different cutoff lengths as indicated in the legend and $(A, \kappa D) = (1.0, 0.5)$(a), $(16.0, 0.5)$(b), $(1.0, 2.0)$(c), $(16.0, 2.0)$(d). The hard rod aspect ratio is $L/D + 1 = 6$ and the effective diameter ratio D^{eff}/D is indicated in the legend.

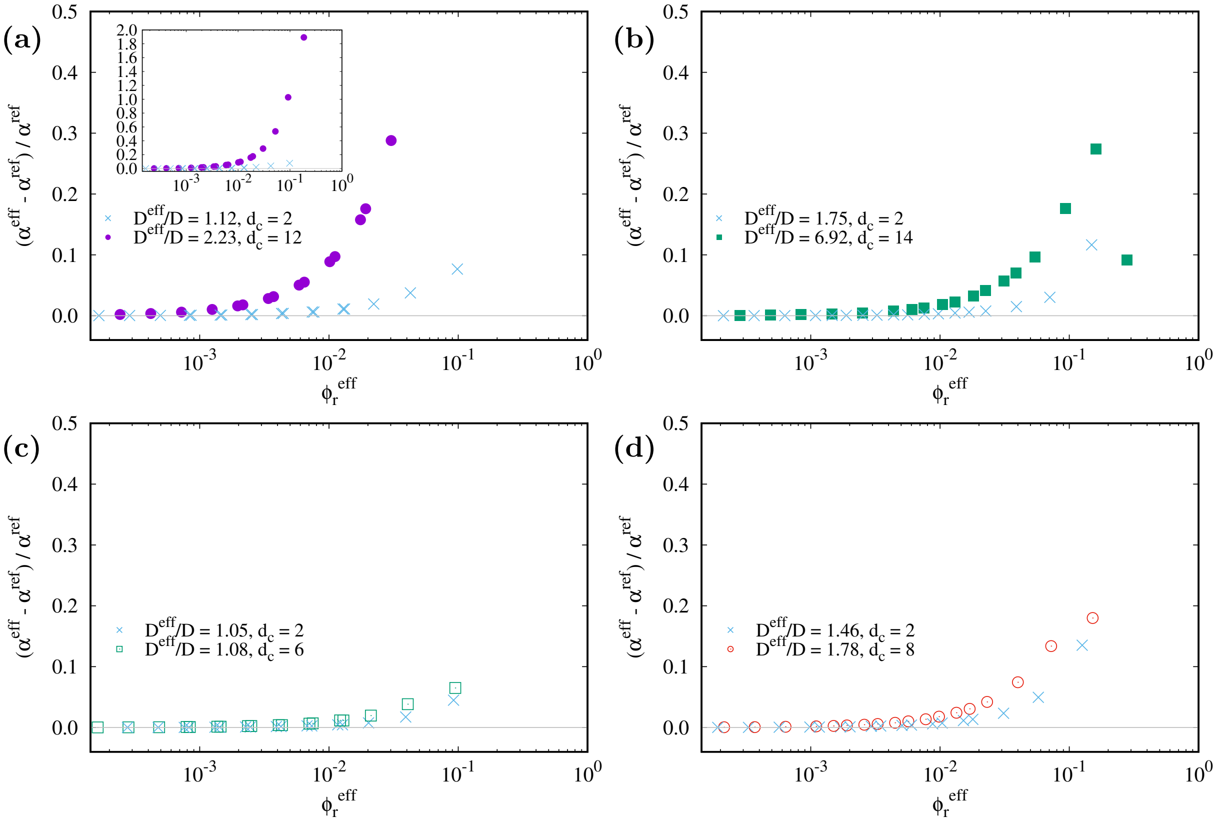

Figure S3: Relative difference between the effective free volume fraction and the reference from the immersion free energy vs. effective volume fraction for different cutoff lengths as indicated in the legend and $(A, \kappa D) = (1.0, 0.5)$(a), $(16.0, 0.5)$(b), $(1.0, 2.0)$(c), $(16.0, 2.0)$(d). The hard rod aspect ratio is $L/D{+}1 = 21$ and the effective diameter ratio D^{eff}/D is indicated in the legend.

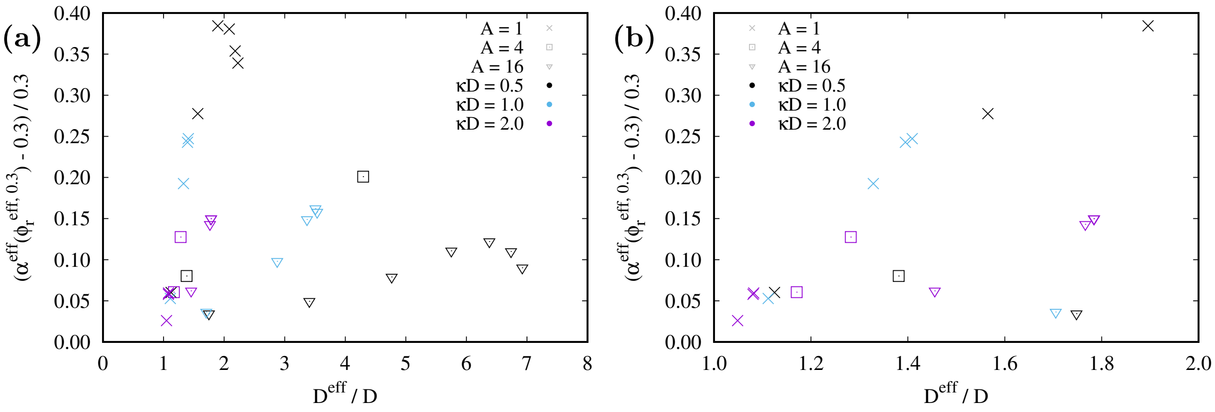

Figure S4: Relative difference between the effective free volume fraction and the reference from the immersion free energy at the effective volume fraction for which the reference equals 0.3 vs. effective diameter for different A and κD. Identical symbols refer to the same $(A, \kappa D)$ but different cutoff lengths (resulting in different effective diameter ratios. The hard rod aspect ratio is $L/D + 1 = 6$. (a) full range of effective diameters. (b) small effective diameters.

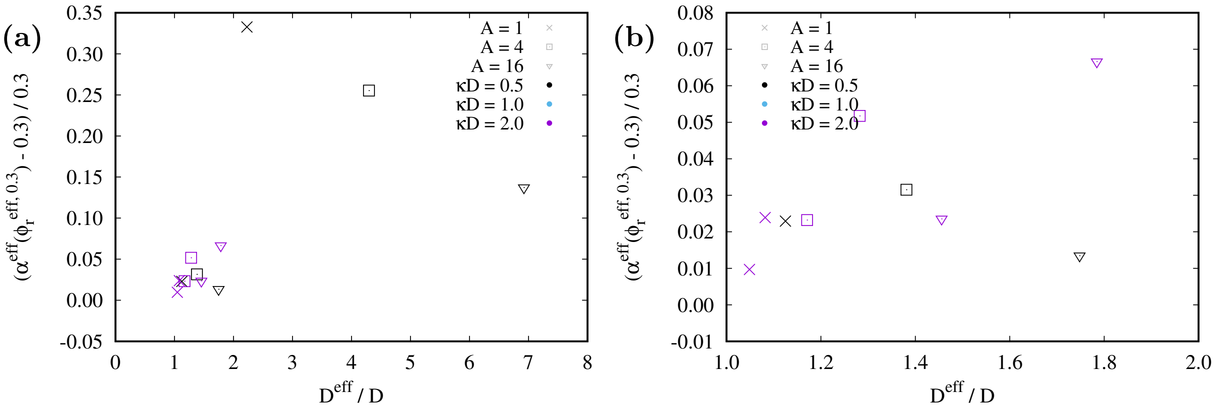

Figure S5: Relative difference between the effective free volume fraction and the reference from the immersion free energy at the effective volume fraction for which the reference equals 0.3 vs. effective diameter for different A and κD. Identical symbols refer to the same $(A, \kappa D)$ but different cutoff lengths (resulting in different effective diameter ratios. The hard rod aspect ratio is $L/D + 1 = 21$. (a) full range of effective diameters. (b) small effective diameters.
